# Transforming mental health implementation research

**DOI:** 10.1192/j.eurpsy.2025.118

**Published:** 2025-08-26

**Authors:** E. McGinty

**Affiliations:** Health Policy & Economics, Weill Cornell Medicine, New York, United States

## Abstract

**Abstract:**

Slow and inadequate implementation causes greater inequity and reduced quality in emergency psychiatric services. This symposium is based on five recommendations to improve health intervention research and implementation, presented by Professor Emma Mcginty (PhD), the leader of the Lancet Psychiatry Commission to transform implementation in mental health. This presentation will introduce each of these recommendations: I) integrate research into clinical practice to ease implementation II) embed equity in mental health intervention research III) use complexity science and bottom-up approaches to improve relevance of science and implementation IV) base implementation less additional methods beyond traditional RCT-based research V) use a transdisciplinary approach in development and implementation of research on new interventions.

**Disclosure of Interest:**

None Declared

